# Evaluation of micronuclei in oral mucosa of individuals exposed to ionizing radiation: a pilot study from Celaya, México

**DOI:** 10.5195/cajgh.2019.331

**Published:** 2019-05-10

**Authors:** Nicolas Padilla-Raygoza, María del Rocio Adame Gutiérrez, Itza Zelene Moreno Martínez, Vicente Beltran-Campos, Silvia del Carmen Delgado-Sandoval, Maria de Lourdes Garcia-Campos, Modesto Antonio Sosa-Aquino, Teodoro Cordova-Fraga, Rafael Guzman-Cabrera

**Affiliations:** 1 Department of Nursing and Obstetrics, Division of Health Sciences and Engineering, Universidad of Guanajuato Mutualismo, México; 2 Academic Unity of Nursing N°1, University Autonomous of Guerrero, México; 3 Division of Health Sciences and Engineering, University of Guanajuato, México; 4 Department of Clinical Nursing, Division of Health Sciences and Engineering, University of Guanajuato, México; 5 Department oh Physical Engineering, Division of Sciences and Engineering, University of Guanajuato, México; 6 Departmentof Electrical Engineering, Division of Engineerings, University of Guanajuato, México

**Keywords:** Ionizing Radiation, Micronuclei, Genetic Damage

## Abstract

**Introduction:**

Occupational exposure to ionizing radiation can potentially lead to adverse health effects, including cancer and genetic defects. Genetic damage caused by radiation can be detected if micronuclei are observed. The objective of this pilot study was to detect the presence of micronuclei in cells of the oral mucosa in inidividuals occupationally exposed to ionizing radiation.

**Methods:**

We implemented a pilot case-control study in which we compared oral mucosa micronuclei in 30 medical and nursing personnel in radiology centers in Celaya, Mexico, with 30 volunteers not exposed to ionizing radiation recruited from a public University. The oral mucosa was brushed and the amount of micronuclei was quantified. Chi-square test or t-test for two proportions were used to compared ionizing radiation and genetic damage between exposed and non-exposed groups.

**Results:**

The exposed group had an average of 5.37 ± 3.49 micronuclei and the non-exposed had 0.37 ± 0.61 (P<0.01). In the exposed group, 90% of participants exhibited genetic damage compared to 6.67% in the unexposed group (P<0.05).

**Conclusion:**

In this pilot study, medical and nursing staff from radiology centers presented with higher genetic damage compared to control group. Further studies are needed to identify the prevalence of genetic damage due to occupational radiation exposure in Mexico.

## Introduction

Chronic exposure to ionizing radiation can potentially lead to significant negative health effects, including increased incidence of cancer as well as genetic and immunological defects^1^. Moreover, exposure to low doses of radiation (less than 50 mSv/year) early in life increases susceptibility to damage from high-dose radiation exposure later in life^2^. Studies on ionizing radiation have been predominantly focused on the high-dose radiation exposure, such as nuclear bombs and nuclear plant spills^1^. The effects of chronic occupational low-dose radiation exposure received little attention.

Many countries have adopted the International Commission on Radiological Protection (ICRP) recommendations of a 20-mSv-per-year occupational effective dose limit with allowances to go as high as 50 mSv per year, as long as the average annual dose over five years does not exceed 20 mSv^3^. However, workers may be exposed to much higher doses during routine operations due to the nature of their occupation.

Micronuclei (MN) are one way of detecting genetic damage caused by radiation. Damage from the radiation leads to the incorrect incorporation of genetic material into the nuclei of the daughter cells, resulting in chromosomal losses and unequal distribution of the genetic material. These genetic aberrations cause the emergence of MN^4^,^5^, small extranuclear bodies that contain centric or acentric chromatid, chromosomal fragments, or complete chromosomes that are not included in the daughter nuclei during mitosis. Thus, MN are cellular markers of chromosomal damage^4^,^6^–^8^. Since buccal epithelial cells reported to be sensitive to radiation, observing MN presence in these cells can be used to accurately monitor potential exposure^9^,^10^. The MN testing has been used to identify early genotoxic effects, as well as to evaluate occupational exposure to mutagenic substances^11^. Since the MN test is non-invasive, individuals are more willing to be screened through this method.

The Mexican Official Norm NOM-229-SSA1-2002 Environmental health, establishes the dose limit for individuals working in the radiology centers at 50 mSv per year^12^. For individuals who do not work in the radiology centers, the annual limit is 5 mSv. Little research has been done on occupation radiation exposure in Mexico, a research gap this study is aiming to fill. The objective of this pilot study was to measure the presence of MN in personnel exposed to ionizing radiation compared to non-exposed personnel in Celaya, Mexico. The hypothesis was that personnel exposed to radiation will have higher MN compared to non-exposed individuals.

## Methods

### Participants

This was a case-control pilot study in which subjects exposed to ionizing radiation were compared to a group of unexposed individuals. Research participants were assessed at the Life Style Laboratory of the Division of Health Sciences and Engineering, Celaya Salvatierra Campus of the University of Guanajuato in Celaya, Mexico. The inclusion criteria for exposed group were full-time employment at public or private x-ray center who reported direct involvement with x-ray equipment. Research participantes included physicians, nurses, and secretaries). The unexposed group consisted of nursing students, nurses, and medical doctors recruited from the University of Guanjuato who did not have regular exposure to x-ray and lived in the same city as the exposed group. The exclusion criteria for both the exposed and unexposed groups were any history of cancer diagnosis and/or presence of acute infectious disease (such as influenza) at the time of oral scraping.

The project was approved by the Bioethics Committee (registration CBDCSI-87141126) through the Division of Health Sciences and Engineering of the Celaya Salvatierra Campus, University of Guanajuato, Mexico.

### Data collection

Information on demographic factors, including age, gender, residence, marital status, and education, was collected using interviewer administered survey. Detailed information about occupational history, including previous experience in x-ray rooms and duration of previous employment, was also collected. Smoking was assessed as a dichotomous categorical variable and was defined as smoking two or more cigarettes a day. Radiation was measured as 1-month uptake of ionizing radiation using a dosimeter. The dosimeters used in this study consisted of one pair of thermoluminescent crystals chips (TLD-100, ThermoFisher Scientific, Waltham, MA, USA) were placed in appropriated badges as previously described^13^. Before use, TLDs were annealed to 400°C for one hour and to 100°C for two hours in a muffle in order to erase any environmental or spurious signals. The badges were worn by the participants on the upper left side of the chest during work hours as the highest radiation exposure is expected in this part of the body^14^. The dosimeter readings were performed using the Harshaw TLD 3500 equipment with appropriated readings parameters.

All participants were given a dosimeter to carry during work hours for a month to measure exposure to ionizing radiation. After a month, the dosimeters were collected and sent for analysis to the Medical Physics Academic Body and Biomedical Instrumentation (León, Mexico). Radiation was defined as high exposure ( ≥4.1 mSv) or low exposure ( ≤4.0 mSv) based on the Official Mexican Norm standards^12^.

Genetic damage was determined as the number of micronuclei present in the oral samples. Oral samples were taken during the study enrollment by brushing the cheek of the mouth and storing the samples in a neutral pH buffer at 10°C until processing, fixation, staining with Giemsa, and second fixation according to the technique previously described by Thomas et al^15^. MN were then quantified by clear field microscopy at 100× magnification and were defined as the number of cells with micronuclei per 1000 cells. Genetic damage was defined as ≥2 MN per 1000 cells and no genetic damage was defined as <2 MN per 1000 cells.

### Statistical analysis

The distribution of categorical variables, including gender, residence, marital status, education, occupation, and smoking, was compared between exposed and non-exposed groups using Chi-square test or t-test for two proportions. The continuous age variable was compared between exposed and non-exposed groups using Student’s t-test. The frequency of high- and low-radiation exposure (≥4.1/month or <4.1 mSv/month) and presence of genetic damage (≥2 or <2 MN per 1000 cells) were represented using percentages and compared between exposed and non-exposed radiation groups using Fisher’s exact test. A two-tailed *P* value of 0.05 or less was considered to be significant. All statistical analyses were performed using STATA 13.0® (Stata Corp., College Station, TX, USA).

## Results

We recruited 30 participants who worked in the x-ray centers (exposed group) and 30 participants who did not work in the x-ray center (non-exposed group). The distribution of the sociodemographic variables for the exposed and non-exposed groups is shown in Table 1. Both groups were predominantly female (53.33% vs 73.30%, *P* >0.05), urban residents (86.67% vs 70.00%, *P* >0.05), smokers (86.67% vs 73,33%, *P*=0.2), and married (50% vs 16.67%, *P*=0.02). The mean age and time at work were higher in the exposed than non-exposed group (*P* <0.01). In the exposed group, there was a mean of MN 5.37±3.49 and in the non-exposed group there was a mean 0.37±0.61 (t-test=−7.73, df 58, *P*<0.01).

High radiation exposure (≥4.1mSv) was significantly more common among the exposed group compared to the non-exposed group (13.33% vs 0.00%, *P*<0.05) (Table 2). Genetic damage (≥2 MN per 1000 cells) was more common among the exposed group compared to the non-exposed group (90.00% vs 6.67%, *P*<0.05) in this sample.

## Discussion

We found a significant difference in MN count between radiation exposed and non-exposed groups in Celaya, Mexico. The exposed group had higher number of participants with unsafe level of exposure (13% vs 0%) based on the Official Mexican Standard -SSA1-2002, Environmental Health^12^.

Our results are consistent with previously published literature. Qian et al.,^16^ found that personnel working with x-rays have a higher MN count than the control group of healthy adults without history of expose to radiation, which similar to the results found in this study, where the exposed group had a higher MN number per 1000 cells compared to the non-exposed group (Table 2). We have also found that individuals working in x-ray centers have been exposed to more radiation than permitted by national guidelines.

Kanaragaj et al.,^17^ quantified the presence of MN in binucleated cells (lymphocytes from peripherical blood) in subjects before and after undergoing computed tomography and found a significant increase in MN after the procedure. Their findings demonstrate that acute exposure to ionizing radiation causes genetic damage. In our study, we measured chronic occupational radiation exposure and found the number of MN in oral cells was significantly higher (*P*<0.05) in those exposed occupationally to ionizing radiation compared with the non-exposed group.

The key limitation of this study is its small sample size. However, this was a pilot study designed to inform future larger investigations. Another limitation is that there was an age difference between exposed and non-exposed group, which may have influenced findings. Also, the measurement of radiation exposure was performed over the course of one month, which may not be sufficient. Another limitation is that there are other methods to identify genetic damage that could provide more insight, such as screening for micronuclei in lymphocytes.

The strength of our study is that the measurement of exposure to ionizing radiation was performed in a consistent way, using standard dosimetry techniques.

The results of this study warrant careful interpretation. While our results show significant differences for markers of genetic damage, the exposed and non-exposed groups are somewhat different in terms of age and several other characteristics to make definitive conclusions. Future studies should concentrate on establishing more robust methodology, recruiting more comparable study populations, as well as improving the sample size. Exposure to ionizing radiation may have a significant effect on genetic damage in individuals, therefore it is important to implement protective measures. In Mexico and other countries, it is very important to provide concise guidelines on planning, performing, and interpreting studies to monitor groups or individuals exposed to genotoxic agents.

## Figures and Tables

**Table 1:** Sociodemographic characteristics by group

Variable	Exposed group		Non-exposed group		*P*-value
	n	%	n	%	
Gender					>0.05
Male	14	46.67	8	26.67	
Female	16	53.33	22	73.33	
Residence					>0.05
Urban	26	86.67	21	70.00	
Suburban	1	3.33	6	20.00	
Rural	3	10.00	3	10.00	
Marital status					<0.01*
Single	9	30.00	25	93.33	
Married	15	50.00	5	16.67	
Separated	1	3.33	0	0.00	
Widowed	2	6.67	0	0.00	
Divorced	1	3.33	0	0.00	
Free union	2	6.67	0	0.00	
Education					<0.01*
Elementary	2	6.67	0	0.00	
Secondary	1	3.33	1	3.33	
High School	3	10.00	1	3.33	
Technical career	15	50.00	0	0.00	
Bachelor degree	2	6.67	26	86.67	
Postgraduate	7	23.33	2	6.67	
Occupation					<0.02*
Receptionist	2	6.67	1	3.33	
Assistant	2	6.67	0	0.00	
Technician	15	50.00	2	6.67	
Nurse	5	16.67	15	50.00	
Medical Doctor	4	13.33	7	23.33	
Support team	2	6.67	5	16.67	
Smoking					0.20
Yes	4	13.33	8	26.67	
No	26	86.67	22	73.33	
Age(years)					
Mean ± S	37.80 ±11.59		25.30 ± 7.09		<.01¥
Time at the work (years)					
Mean±S	10.83±7.86		2.08±2.49		<.01¥

* Fisher’s Exact-Test

¥ Student’s t test

**Table 2:** Distribution of exposure to ionizing radiation and genetic damage by the exposure group

	Exposed group (n=30)		Non-exposed group (n=30)		P-value
	f	%	f	%	
Exposure to radiation (mSv/month)*					
High (≥4.1)	4	13.33	0	0.00	0.04*
Low (<4.1)	26	86.67	30	100.00	
Number of Micronuclei (per 1000 cells)					
Genetic damage	27	90.00	2	6.67	<.01
Non-genetic damage	3	10.00	28	93.33	

* Fisher Exact Test
